# Natural Emulsions Based on Essential Oils as Antifungal and Antimycotoxicogenic Agents on Wheat for Bakery Industry

**DOI:** 10.3390/foods11182926

**Published:** 2022-09-19

**Authors:** Ersilia Alexa, Voichita Bota, Renata Maria Sumălan, Diana Obistioiu, Monica Negrea, Ileana Cocan, Florin Borcan, Antoanela Cozma, Isidora Radulov

**Affiliations:** 1 Faculty of Food Engineering, Banat’s University of Agricultural Sciences and Veterinary Medicine, “King Michael I of Romania” from Timisoara, Calea Aradului No. 119, 300645 Timisoara, Romania; 2 Faculty of Horticulture and Forestry, Banat’s University of Agricultural Sciences and Veterinary Medicine, “King Michael I of Romania” from Timisoara, Calea Aradului No. 119, 300645 Timisoara, Romania; 3 Faculty of Veterinary Medicine, Banat’s University of Agricultural Sciences and Veterinary Medicine, “King Michael I of Romania” from Timisoara, Calea Aradului No. 119, 300645 Timisoara, Romania; 4 Faculty of Pharmacy, “Victor Babeş” University of Medicine and Pharmacy, Eftimie Murgu Sq. No. 2, 300041 Timişoara, Romania; 5 Faculty of Agriculture, Banat’s University of Agricultural Sciences and Veterinary Medicine, “King Michael I of Romania” from Timisoara, Calea Aradului No. 119, 300645 Timisoara, Romania

**Keywords:** deoxynivalenol (DON), *Thymus vulgaris*, *Origanum sativum* and *Coriandrum sativum*, emulsions

## Abstract

This study aimed to investigate the antifungal and antimycotoxicogenic effect of binary and tertiary mixtures of *Thymus vulgaris*, *Origanum sativum*, and *Coriandrum sativum* essential oils (EOs), as well as emulsions based on EO mixtures, on fungi developed on wheat grains destined for the bakery industry. The chemical composition of the EO mixtures, the physical characteristics of the emulsions, and the influence of treatments on the proximate composition of wheat seeds were also studied. The methods used included the microbiological analysis of fungi developed on wheat seeds, the ELISA technique for determining the deoxynivalenol content (DON), gas chromatography coupled with mass spectrometry (GC-MS) to detect the chemical composition of the EOs, Zetasizer to analyse the particle sizes and their electric charge at the surface, and NIR analysis of the proximate composition of wheat. The chemical composition analysis revealed that thymol and o-cymene were the major components in the binary mixture of the EOs with thyme, linalool in the binary mixtures of the EOs with coriander and carvacrol, and o-cymene in the binary mixtures of the EOs with oregano. The results showed that, based on the zeta potential, the tertiary mixture ensured maximum emulsion stability, while the emulsion based on thyme and oregano was the less stable system. Regarding the antifungal and antimycotoxicogenic effect, the results showed that the highest inhibition potential on fungi was observed with the binary mixtures of the EOs based on thyme and oregano, and on deoxynivalenol (DON) when the binary emulsion based on the same EOs was applied to wheat seeds. The proximate composition of wheat seeds contaminated with DON showed an increase in protein content and mineral substances, and there were changes in the colour of the wheat seeds after treatment with the EOs. In conclusion, the results obtained in this study showed the possibility of using binary/tertiary mixtures of EOs and emulsions as healthy and environmentally friendly alternatives in the bakery industry.

## 1. Introduction

According to statistics, cereals and cereal products are one of the most important sources of food for humans and animals. Annually, a significant part of cereal production is affected by fungal attacks in the field or during storage, causing significant losses from an economic point of view but also negative effects on human and animal health [1]. The current trend of consumers choosing safe and healthy foods has increased interest in organic farming. Organic products have grown considerably in the European market, with almost one million hectares in recent years. According to the Organic Agriculture Research Institute (FIBL), the country with the largest organic agricultural area is Spain (1.3 million hectares), followed by Italy (1.1 million hectares), and Germany (0.95 million hectares), with Italy being the major exporter of organic products in Europe [2].

In the absence of fungicides traditionally applied in conventional agriculture, fungi affect cereals as potential producers of several mycotoxins in organic farming. These moulds colonise many important foods and predominate in warm and temperate areas, mainly in central and southeastern Europe. Fusarium sp., especially *Fusarium graminearum* and *Fusarium culmorum,* are the fungi that occur in cereals and produce mycotoxins [3,4]. The incidence of mycotoxin contamination is widespread, and it is estimated that 25–60% of grain production is contaminated with mycotoxins [5]. In cereals, fungal contamination leads to the development of mycotoxins from the trichothecene group. Deoxynivalenol (DON, also called vomitoxin) is the most common trichothecene developed in wheat and wheat-based products. The incidence of DON in wheat and wheat products is very high and depends on the climate, field conditions, and the temperature and humidity of grain warehouses. Igbal et al. 2020 reported that 44.8% of wheat samples and products from the summer season and 41.9% of samples from the winter season were contaminated with DON [6].

Deoxynivalenol frequently contaminates cereals and cereal products and occurs both in cereals and in floury foods, posing a serious threat to health because it affects the immune and gastrointestinal systems of humans and animals, causing digestive disorders [7]. The European Union (EU) population is frequently exposed to DON, mainly due to the consumption of bread and other bakery products [8].

The accumulation of mycotoxins in grains and the consumption through bread or bakery products in considerable doses can pose a potential risk to human and animal health. Natural measures to prevent/reduce and combat mycotoxinogenesis and reduce the consumption of chemicals in the food industry are being studied to discover and develop new alternative strategies to ensure food security and environmental protection [9].

In stored cereals, applying natural preservatives and EOs inhibits the production of mycotoxins [10]. Moreover, the combination of chemical compounds and natural products can generate a 90% reduction in DON biosynthesis [11].

Previous studies have investigated different systems to obtain natural preparations based on EOs with antifungal potential in order to select the most suitable EO, carrier, and/or emulsifier for preparing the emulsion. Bacterial cellulose nanocrystals (BCNCs)/fish gelatin (GelA)-based emulsion [12], chitosan coatings and films loaded with different EOs [13,14], EOs encapsulated in lecithin [15,16], low-density polyethylene [17], octenyl succinic acid starch [18], Tween [19], zeolite, gelatin [20], and nanocellulose [21] have been tested. Significant results have been obtained by incorporating EOs into different coarse emulsions (CEs), nanoemulsion (NE), and Pickering emulsion (PE) at different concentrations [17].

Although the antifungal and antibacterial potential of EOs have been demonstrated [22], there are currently few studies on the synergism or antagonism exerted by the shared use of natural preparations of emulsions based on EOs.

Previous studies have shown the antifungal potential of EOs extracted from *Origanum sativum* (oregano), *Coriandrum sativum* (coriander), and *Thymus vulgaris* (thyme) [6,23]. The fungicidal effect of these EOs, their potential to control storage fungi, and the prevention of DON mycotoxins in cereals and cereal products were argued [24].

Based on our previous results that demonstrated the antifungal and antimycotoxicogenic potential of coriander, thyme, and oregano EOs [24], in this study we aimed, on the one hand, to evaluate the synergistic/antagonistic effects of combining previously analysed EOs and, on the other, to obtain natural emulsions based on binary and tertiary mixtures of EOs and to test them in terms of their antifungal and antimycotoxicogenic effects.

This study aimed to investigate natural preparations based on binary/tertiary mixtures of EOs with antifungal and antimycotoxicogenic roles in order to offer alternative solutions to the synthetic compounds used in grain preservation, thus ensuring food safety and significantly reducing the side effects of mycotoxins on human health.

In this regard, the objectives of this study included: (i) obtaining binary and tertiary mixtures based on EOs; (ii) a chemical characterisation of the mixtures by GC-MS; (iii) obtaining and characterising natural emulsions based on EOs; (iv) evaluating the antifungal and antimycotoxicogenic potential of EO mixtures and natural emulsions on wheat grains destined for the bakery; and (v) studying the influence of fungal/mycotoxin contamination on the physical–chemical properties of wheat flour.

## 2. Materials and Method

### 2.1. The Obtaining of EO Mixtures and Natural Preparations

The EOs used in the study were purchased from SOLARIS PLANT SRL, Bucharest, Romania. The EO mixtures were obtained according to Table 1.

### 2.2. Chemical Composition of EO Mixtures

The chemical composition of the EOs was determined using a GS/MS QP 2010 Plus (Shimadzu, Kyoto, Japan) equipped with an AT WAX 30 m × 0.32 mm × 1 μm capillary column (Santa Clara, CA, USA). The temperature program to separate the compounds was: 40 °C/min, followed by increasing the temperature to 210 °C with a rate of 5 °C/min and holding for 5 min. Helium was used as a carrier gas at a rate of 1 mL/min; injector and ion source temperatures were 250 °C and 220 °C, respectively. The injection volume was 1 μL at a split ratio of 1:50. To identify the volatile compounds, the NIST 02 and Wiley 275 spectra libraries were used. The results are expressed as a relative percentage of total compounds. All analyses were performed in duplicate.

### 2.3. Preparation and Characterisation of Natural Emulsions

The natural emulsions based on EOs were prepared as an oil-in-water (O/W) system using lecithin as the emulsifying agent [25]. The composition of the emulsions is presented in Table 1. The concentration of the EOs was selected based on an extensive literature search and several experimental tests previously conducted by our group [24,26]. The composition of the emulsions was determined after establishing the minimum concentration with fungistatic effect (CMFs) and the minimum concentration with fungicidal effect (CMFg) of the EOs against *Fusarium graminearum*, the most important fungal pathogen on wheat for bakery products, as described in Section 2.6.

All the ingredients were mixed using an Ultrasonic Processor VCX130 PB 130 Watt, Frequency 20 kHz, (Sonics&Materials INC., Newtown, CT, USA) for 10 min at an amplitude of 98%.

A zetasizer instrument (Cordouan Technology, Cité de la Photonique, France) was used to analyse the particle sizes and their electric charge at the surface. The analysed parameters were: mean particle size (nm), polydispersity index (PDI), and zeta potential (mV). The working parameters were: temperature (23 °C), laser power (80 ± 5%), channel number (~450) and time range (15 ± 5 µs), data acquisition mode—continuous, five measurements at medium resolution.

### 2.4. Establishing the Minimum Concentration with Impact on Mycelial Growth of Fungi

In order to establish the EO concentrations required for the preparation of emulsions, the minimum concentrations with impact on the mycelial growth of fungi were determined by the food poison technique [27]. The culture medium was CYGA (chloramphenicol yeast glucose agar), and the EO concentrations tested were: 0.02%, 0.04%, 0.06%, 0.1%, 0.2%, 0.3%, 0.5%, and 0.6%.

The technique consisted in obtaining 9 mm-diameter mycelial discs from *Fusarium graminearum* 4-day cultures which were harvested and transferred to the variants of the media mixed with different amounts of EOs in Petri plates. Incubation was performed at 22 ± 2 °C in 12:12 h light:dark cycles. At day 5, two perpendicular diameters were measured for each inoculated mycelial disc. The raw data were used to calculate the increased mycelial area (IMA) using the formula:

(1) $$IMA=\frac{\left[\left(\frac{RM^{2}\times 3.14}{4}\right)-IIS\right]}{100}$$

where:

RM—radius of the mycelial surface.
IIS—initial inoculated surface.

The results (cm^2^) were subsequently expressed as a percentage of the control.

### 2.5. Treatment of Wheat Seeds

The seed treatment was performed according to a previous study [24]. The wheat seeds (Antille variety) were washed with 1:9 (*v*/*v*) sodium hypochlorite solution to remove opportunistic microorganisms, and the seeds were rinsed three times with sterile distilled water and dried at 100 °C until a relative humidity of 14%. Then, 300 g of wheat seeds were placed in containers with different variants of EOs and natural emulsions based on EOs. The vapours of the EOs and emulsions were added to a sterile filter paper and inserted into each container with wheat seeds. The containers were kept in the dark and periodically shaked to ensure the homogeneous exposure of the seeds to EO vapours. The experiment was conducted in triplicate.

### 2.6. Analysis of Fungal Contamination

The method used to detect the fungal growth involved the direct plating technique on dichloran rose bengal chloramphenicol (DRBC) medium (Oxoid, CM0727, Thermo Fisher Scientific Inc., Hemel Hempstead, England) [24]. After the exposure of wheat seeds to vapours of different EO mixtures and emulsions, the Petri plates were incubated in the dark at 22 °C for 3–4 days, and then the number of colonised seeds was registered at 7, 14, 21, and 28 days. Fungi were isolated in pure cultures on the 6th day for their identification. Confirmation of the *Fusarium* species was performed using a specific medium, dichloran chloramphenicol peptone agar (DCPA) medium (Sigma-Aldrich Chemie GmbH, Taufkirchen, Germany), with a photoperiod of 12 h light/night. The negative control was prepared in the same way as the samples, using only the solvents without addition of EOs or emulsions.

The frequency of occurrence of each fungal genus from the total fungal genera was calculated using the formula:

(2) $$Fr\left(\%\right)=\frac{NG}{TNF}\times 100$$

where:

*NG* = number of genera.
*TNF* = total number of fungi in the sample.

### 2.7. Analysis of Mycotoxin Content

The wheat samples (control and treated with the EO mixtures and emulsions) were grounded using a laboratory mill (Grindomix Retsch GM 2000, Haan, Germany). The samples (5 g) were homogenised for 20 min with 100 mL of distilled water using a stirrer (I.D.L., Freising, Germany). The extracts were filtered, and 1 mL of the filtrate was used for DON analysis based on enzyme-linked immunosorbent assay (ELISA) using R-Biopharm kits (Bio-Rad Laboratories Redmond, WA-USA). The procedure was followed according to the manufacturer’s instructions. The absorbance of extracts was measured at 450 nm using an ELISA 96-well plate reader (PR-1100, Bio-Rad Laboratories, Hercules, CA, USA). The results are expressed in ppm. The negative control was prepared in the same way as the samples, using only the solvents without the addition of EOs or emulsions. Standards of different concentrations of DON were used for quantitative analysis.

The DON inhibition percentage (%) was evaluated using the formula:

(3) $$Inhibition\;\left(\%\right)=\frac{IC-FC}{IC}\times 100$$

where:

IC—initial DON content in wheat seed without treatment (ppm).
FC—final DON content in wheat seed after treatment with different EO mixtures and natural preparations (ppm).

### 2.8. Physical–Chemical Analysis of Wheat Seeds

The ground samples were used for the physical–chemical analysis of wheat seeds. The proximate composition of wheat seeds was analysed using the NIR grain analyser, Inframatic 9500 (Perten Instruments AB, Hägersten, Sweden). The monochromator was precalibrated for moisture, protein, ash, and colour. The analysis was performed according to the manufacturer’s recommendation; the sample size was 400 mL and the time of analysis was 25 s. The instrument was standardised to the NIST wavelength standard.

### 2.9. Statistical Analysis

All determinations were carried out in triplicate, and the results are reported as mean value ± standard deviation (SD). Antimicrobial activity rates, chemical data, figures, and statistical correlation were recorded with Microsoft Excel 365 (Version 2208, Redmond, WA, USA). 

## 3. Results

### 3.1. Chemical Composition of EO Mixtures

Table 2 displays the chemical composition of the binary and tertiary mixtures of the EOs.

The major compounds in the binary mixture TO were: o-cymene (33.25%), thymol (43.65%), and carvacrol (30.35%). The TC mixture presented a high percentage of o-cymene (29.33%), β-linalool (28.87%), and thymol (26.18%) as the major components, while the OC binary mixture was characterised by o-cymene (24.35%), β-linalool (28.22%), and carvacrol (35.72%).

### 3.2. Physical Characterisation of Natural Preparations

The mean physical parameters of the obtained emulsions are presented in Table 3.

The results from Table 3 showed that, except for TCE, a unipopulational emulsion (100% proportion of the population) with a mean particle size of 723.9 nm, the other emulsions were characterised by two-particle populations with different proportions: (i) OCE with 23% of particles with a mean size of 617.1 nm and 77% of particles with a mean size of 883.4 nm; (ii) TOE with 48% of particles with a mean size of 583.3 nm and 52% of particles with a mean size of 701.8 nm; (iii) TOCE with 63% of particles with a mean size of 570.7 nm and 37% of particles with a mean size of 887.6 nm.

### 3.3. Fungal Contamination

**Figure 1:**
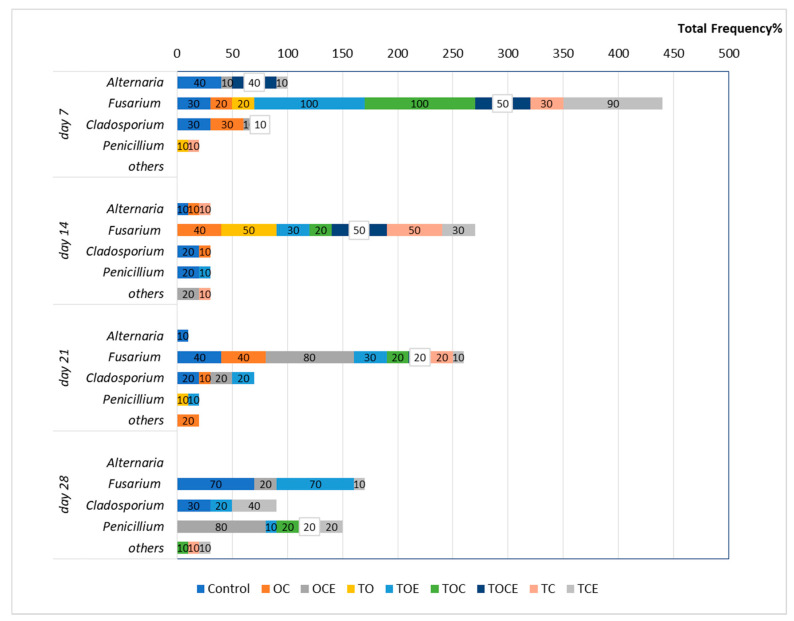
The total fungi frequency (Fr%) after 7, 14, and, 28 days of treatment with EOs and natural preparations. OC—binary mixture of oregano and coriander EOs; OCE—binary emulsion of oregano and coriander EOs; TO—binary mixture of thyme and oregano EOs; TOE—binary emulsion of thyme and oregano EOs; TC—binary mixture of thyme and coriander EOs; TCE—binary emulsion of thyme and coriander EOs; TOC—tertiary mixture of oregano, coriander, and thyme EOs; TOCE—tertiary emulsion of oregano, coriander, and thyme EOs.

Figure 1 presents the wheat seeds’ fungal contamination, expressed as the total frequency (Fr %) after EO and emulsion exposure at different time intervals (7, 14, 21, and 28 days).

From the results presented in Figure 1, it can be observed that after 7 days of fumigation the following fungal species are found in the control treatment: *Alternaria* (Fr = 40%), *Fusarium* (Fr = 30%), and *Cladosporium* (Fr = 30%). After 14 days, the fungal incidence decreased to 10% for *Alternaria* and 20% for *Cladosporium* and *Penicillium*. After 21 days, *Alternaria* was found with Fr = 10%, *Fusarium* Fr = 40%, and *Cladosporium* Fr = 20%, while after 28 days, *Fusarium* was observed with Fr = 70%.

**Figure 2:**
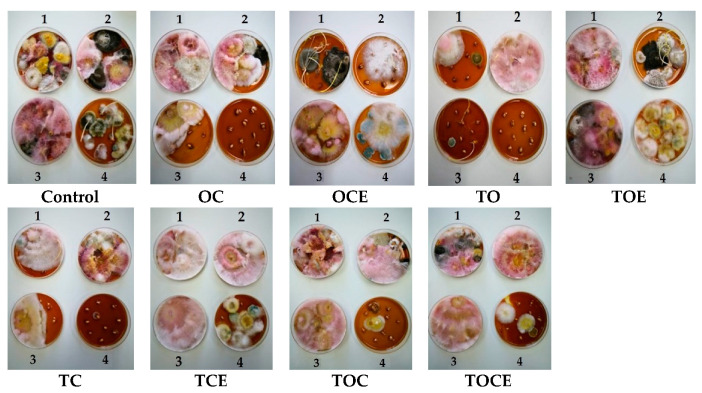
The appearance of mycelium development after exposure to EOs and emulsions for 7 days of treatment (1), 14 days (2), 21 days (3), and 28 days (4). OC—binary mixture of oregano and coriander EOs; OCE—binary emulsion of oregano and coriander EOs; TO—binary mixture of thyme and oregano EOs; TOE—binary emulsion of thyme and oregano EOs; TC—binary mixture of thyme and coriander EOs; TCE—binary emulsion of thyme and coriander EOs; TOC—tertiary mixture of oregano, coriander, and thyme EOs; TOCE—tertiary emulsion of oregano, coriander, and thyme EOs.

Regarding the samples with antifungal treatment, after 7 days of fumigation the development of *Fusarium* can be observed in seven experimental trials (Figure 2). Hence, the exposure to the TOE and TOC vapours increased *Fusarium* growth relative to the control, with FR = 100%, as well as FR = 90% for TCE. In addition, TOCE influenced the development of *Fusarium* (Fr = 50%). Lower development was observed after exposure to the OC, TC, and TO vapours (Fr = 20–30%). Excepting the treatments with OC (Fr = 30%), OCE (Fr = 10%), and TOCE (Fr = 10%), no development of *Clodosporium* was observed. *Alternaria* was inhibited except when OCE, TCE, and TOCE were applied to wheat seeds. *Penicillium* was observed in one sample with low frequency (10% for TC).

After 14 days of treatment, *Fusarium* exhibited an increase in mycelium in the case of OC (Fr = 40%), TO, and TC (Fr = 50%), while the use of TOE, TOC, and TCE led to a decrease in mycelium growth (Figure 2). *Cladosporium* was observed only in the control and in OC, while the development was inhibited in the trials with other EO and emulsion treatments. *Alternaria* was also sensitive to treatment with EOs and emulsions, with mycelium growth only after TC treatment (Fr = 10%).

After 21 days of treatment, the pattern of fungi developed on the seeds was similar to that obtained after 14 days for most of the experimental variants. Excepting TO, *Fusarium* was identified in all trials.

After 28 days, the exposure of wheat seeds to EO and emulsion vapours led to the full inhibition of *Fusarium* mycelium when TO, OC, TOC, TC, and TOCE were used (Figure 2). Only TOE, TCE, and OCE allowed *Fusarium* development. Binary emulsions (TO, TC, and OC) had maximum antifungal potential after 28 days against all the analysed pathogens (Figure 2). Notably, after 28 days of treatment *Penicillium* sp. was developed in most samples but not in the control.

### 3.4. Minimum Concentration with Impact on Mycelial Growth of Fungi

The minimum concentration with fungistatic effect (CMFs) is the concentration at which the fungal growth is zero (SMN = 0) and the inoculated fungal disc is transferred to another fresh culture medium (without the addition of EOs) where the resumption of the hyphal growth and the restoration of the fungal mycelium occurs.

The minimum concentration with fungicidal effect (CMFg) is the concentration at which the fungal growth is zero (SMN = 0) and the inoculated fungal disc is transferred to another fresh environment where the resumption of hyphal growth is not observed and the mycelium is inactivated.

It is noticed (in Table 4) that the most effective EO against *Fusarium graminaerum* is oregano EO, with the lowest CMFs (0.06%) and CMFg (0.2%) values, followed by thyme (CMFs 0.1% and CMFg 0.6%), and coriander (CMFs 0.5% and no CMFg observed).

### 3.5. DON Mycotoxin Content

**Figure 3:**
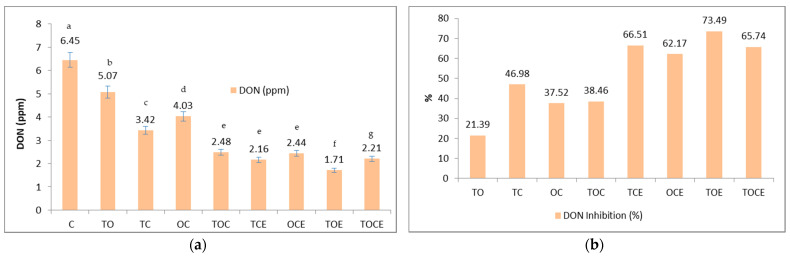
The DON content (**a**) and the percentage of DON inhibition (**b**) after 28 days of treatment with EOs and natural preparations. OC—binary mixture of oregano and coriander EOs; OCE—binary emulsion of oregano and coriander EOs; TO—binary mixture of thyme and oregano EOs; TOE—binary emulsion of thyme and oregano EOs; TC—binary mixture of thyme and coriander EOs; TCE—binary emulsion of thyme and coriander EOs; TOC—tertiary mixture of oregano, coriander, and thyme EOs; TOCE—tertiary emulsion of oregano, coriander, and thyme EOs. The values are expressed as mean values ± standard deviations of all measurements. Different superscripts ^a–g^ between samples represents significantly different values (*p* < 0.05) according to the *t*-test; the same superscripts ^a–g^ between samples represents not significantly different values (*p* > 0.05).

Figure 3 presents the wheat seeds contaminated with DON and the percentage of DON inhibition after 28 days of treatment with EOs and natural emulsions.

Figure 3 shows that the mixtures of EOs and binary and tertiary emulsions have an inhibitory effect on the development of DON. Initially, the DON level in the untreated sample was 6.45 ppm. After the treatment, the DON level decreased, with recorded values of 2.48 ppm in the TOC tertiary samples and 4.03 in the OC binary mixture samples. Regarding the emulsions used, the antimycotoxicogenic effect was more pronounced, with DON values between 1.71 and 2.44 ppm. Expressed as a percentage (Figure 3b), DON inhibition varied in the case of mixtures of EOs in the order: TC > TOC > OC > TO, while in emulsions the order of inhibition was: TOE > TCE > TOCE > OCE. It should be noted that the mixtures that include thyme in the composition showed the maximum inhibition rate.

Regarding DON inhibition (%), the maximum effect was registered when using emulsions (62.17–73.49%) compared to EOs (21.39–46.98%), with TOE emulsion the most effective (73.49%), with a higher inhibition of DON when using emulsions (62.17–73.49%) compared to EOs (21.39–46.98%).

From a statistical point of view, significant differences were identified (*p* < 0.05) among the samples treated with the binary and tertiary mixtures of EOs/emulsions, and the control, between the following pairs: TO/TC, TO/OC, TC/OC, TO/TOC, TO/TCE, TO/OCE, TO/TOE, TO/TOCE, TC/TOC, TC/TCE, TC/OCE, TC/TOE, TC/TOCE, OC/TOC, OC/TCE, OC/OCE, OC/TOE, and OC/TOCE (Figure 3a).

**Figure 4:**
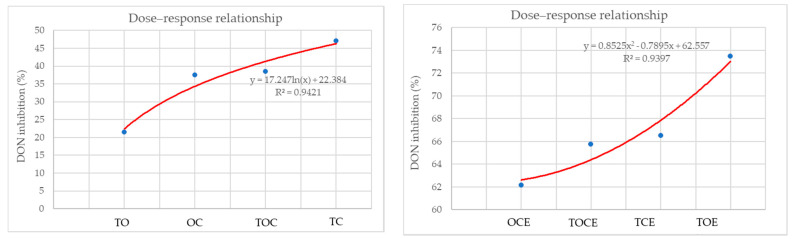
The dose–response relationship between DON inhibition (%) and the percentage of individual EOs used in EO mixtures and emulsions. OC—binary mixture of oregano and coriander EOs; OCE—binary emulsion of oregano and coriander EOs; TO—binary mixture of thyme and oregano EOs; TOE—binary emulsion of thyme and oregano EOs; TC—binary mixture of thyme and coriander EOs; TCE—binary emulsion of thyme and coriander EOs; TOC—tertiary mixture of oregano, coriander, and thyme EOs; TOCE—tertiary emulsion of oregano, coriander, and thyme EOs.

Figure 4 presents the dose–response relationship between DON inhibition (%) and the percentage of individual EOs used in EO mixtures and emulsions. It can be seen that the best correlation in the case of the EO mixtures was obtained for the TC binary mixture and decreases in the order: TC > TOE > OC > TO. For emulsions, there is an increase in the dose–effect dependence in the order: OCE < TOCE < TCE < TOE.

### 3.6. Physical–Chemical Analysis of Wheat Seeds

**Figure 5:**
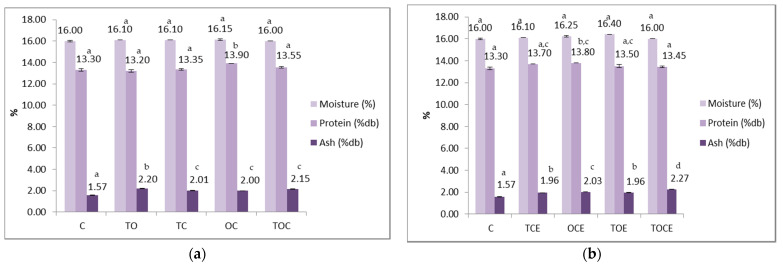
Physical–chemical parameters of wheat seeds contaminated with DON, before and after treatment with: (**a**) binary and tertiary mixtures of EOs, and (**b**) binary and tertiary mixtures of natural emulsions. C—control; OC—binary mixture of oregano and coriander EOs; OCE—binary emulsion of oregano and coriander EOs; TO—binary mixture of thyme and oregano EOs; TOE—binary emulsion of thyme and oregano EOs; TC—binary mixture of thyme and coriander EOs; TCE—binary emulsion of thyme and coriander EOs; TOC—tertiary mixture of oregano, coriander, and thyme EOs; TOCE—tertiary emulsion of oregano, coriander, and thyme EOs. The values are expressed as mean values ± standard deviations of all measurements. Different superscripts ^a–d^ between samples for the same parameters represents significantly different values (*p* < 0.05) according to the *t*-test; the same superscripts ^a–d^ between samples for the same parameters represents not significantly different values (*p* > 0.05).

**Figure 6:**
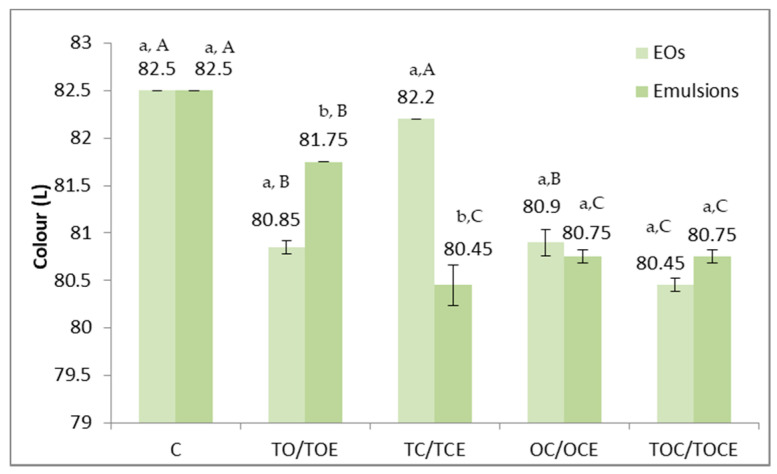
Colour parameters of wheat seeds contaminated with DON, before and after treatment with binary and tertiary mixtures of EOs and natural preparations. C—control; OC—binary mixture of oregano and coriander EO; OCE—binary emulsion of oregano and coriander EOs; TO—binary mixture of thyme and oregano EOs; TOE—binary emulsion of thyme and oregano EOs; TC—binary mixture of thyme and coriander EOs; TCE—binary emulsion of thyme and coriander EOs; TOC—tertiary mixture of oregano, coriander, and thyme EOs; TOCE—tertiary emulsion of oregano, coriander, and thyme EOs. The values are expressed as mean values ± standard deviations of all measurements. Different superscripts ^a,b^ between EOs and emulsions for the same binary or tertiary mixture represent significantly different values (*p* < 0.05) according to the *t*-test; different superscripts ^A–C^ between all types of EOs concerned and between all types of emulsions represent significantly different values (*p* < 0.05) according to the *t*-test.

Physical–chemical parameters of wheat seeds contaminated with DON, before and after treatment with binary and tertiary mixtures of EOs and natural emulsions, are presented in Figure 5. The colour parameters of wheat seeds contaminated with DON are presented in Figure 6.

In the case of the samples with EOs (Figure 5), it is noticed that the humidity remained within tight limits (between 16 and 16.45%), without significant differences between samples.

The protein content in the control sample is 13.3%, indicating that wheat is suitable for the bakery according to the standards in force. In the case of EO treatment, the protein content ranged between 13.2 and 13.9%, the minimum value being recorded for the sample sprinkled with TO and the maximum for the sample with OC. Similar values, between 13.45 and 13.8%, were recorded in the case of treatment with emulsions.

Mineral substance values were higher when applying treatments with EOs and emulsions. The values recorded for EOs were 1.995–2.195%, with significant differences between the control sample (1.57%) and the treated samples. In the case of emulsions, the values were between 1.955 and 2.27%, the maximum being recorded for the tertiary sample (TOCE).

From a statistical point of view, there were no significant differences between the values of humidity recorded for the samples treated with EOs and the control, but there were differences between the values of the control and the samples treated with emulsions, except for the TOCE variant.

Regarding the protein content, there were significant differences (*p* < 0.05) between the sample treated with OC and the other experimental variants, between the control and OCE, and between OCE and TOCE. The content of mineral substances differed statistically between the control and the variants with EO treatment, and between the TO and the other experimental variants. Regarding the emulsions, there are significant differences (*p* < 0.05) between the control and all the experimental variants, but also between TCE/OCE, OCE/TOE, OCE/TOCE, and TOE/TOCE.

The colour parameters of the analysed wheat samples (Figure 6) differed depending on the treatment and the application method. Maximum values (82.5) were recorded in the case of the control and minimum values in the treatment with TEC emulsion, EOs, and TOC (80.45). Statistically significant differences (*p* < 0.05) were recorded between the TO/TOE and TC/TCE pairs. As reported for the control, significant differences in terms of colour were observed for all experimental variants, except TC.

### 3.7. Correlations

Table 5 presents the correlations between DON content and the chemical composition of the binary mixtures of EOs and DON inhibition and the chemical composition of the binary mixtures of EOs. There are moderate to strong correlations (r > 0.7) between the percentage of DON inhibition and the main chemical compounds of the analysed EOs, and a strong negative correlation between the content of DON in the treated samples and the percentage of main chemical compounds in the EOs. The main chemical compounds of the analysed EOs were: o-cymene, β-linalool, thymol, and carvacrol. This analysis showed a strong positive correlation between o-cymene and DON inhibition (r = 0.928), β-Linalool and DON inhibition (r = 0.865), thymol and DON inhibition (0.710), and carvacrol and DON inhibition (r = 0.983)—coefficients that confirm the inhibition potential of these terpenic compounds.

## 4. Discussions

### 4.1. Chemical Composition of EO Mixtures

The chemical composition of the individual EOs of coriander, oregano, and thyme has been extensively studied. However, to our knowledge, there are no studies on the chemical composition of some binary and tertiary mixtures of the EOs analysed or on the natural emulsions based on them.

In previous papers regarding the antifungal and antimycotoxicogenic potential of individual EOs of coriander, thyme, and oregano, it was highlighted the contribution of thymol and o-cymene as the major components in thyme EO, linalool in coriander EO, and carvacrol and o-cymene in oregano EO [24,25,26,27,28,29,30,31,32,33]. Other minor compounds, such as caryophyllene, β-myrcene, *p*-cymene, and γ-terpinene, were identified in the three analysed EOs [34,35,36,37,38,39,40,41].

### 4.2. Physical Characterisation of Natural Emulsions Based on EOs

The emulsion system proposed in the present study is of the oil-in-water type (O/W), in which the EO is the dispersed phase and water is the dispersion medium [25]. As an emulsifier, we used a natural product (lecithin). Emulsifiers can potentiate the antifungal activity of EOs. Some natural emulsifiers, such as soy lysolechitin, enhance the antifungal and mycotoxin inhibitory activities of food-grade thyme oil nanoemulsions, which has been previously demonstrated [42].

The physical stability of emulsion systems is characterised by a mean particle size that describes the average size of the dispersed oil droplets. Generally, the usual particle size for stable emulsions is 200–5000 nm, and the higher the particle size, the more stable the emulsions. An emulsion is fine when the dispersed particles are 250–5000 nm and coarse when the particles are in the range of 5000–10,000 nm. The mean particle size (nm) of our emulsions ranged between 570.7–887.6 nm, falling into the category of stable emulsions. The concentration influences the particle sizes of the emulsion. The study by Wang et al. [18] showed that at a concentration of 5% EO the particle sizes were 0.485 μm and increased to 1.28 μm when the EO concentration was 15%.

The polydispersity index (PDI) measures the size distribution and stability of droplet size in the emulsion [43]. PDI values are between 0 and 1, where 0 is specific to homogeneous systems and 1 to highly heterogeneous ones. TCE has the lowest PDI value (0.3) and is characterised by a monodisperse (unipopulation) delivery system that recommends this formulation as a more stable solution. It is considered that a safe formulation based on a stable and efficient system requires the preparation of a monodisperse (unipopulation) delivery system [44].

The zeta potential is used in colloid chemistry to observe the behaviour of dispersive systems in liquids and characterises the electrical double layer on the solid/liquid interface, which is very important in flotation and flocculation processes [45]. The emulsion’s stability is attributed to forming a thin layer around the EO droplets and is characterised by the zeta potential. Depending on this value, the system can be characterised as having: (i) maximal agglomeration and precipitation (values between 0 and +3 mV); (ii) strong agglomeration and precipitation (values between +5 and −5 mV); (iii) the beginning of agglomeration (values between −10 and −15 mV); (iv) low dispersion (values between −16 and −30 mV); (v) medium stability (values between −31 and −40 mV); (vi) good stability (values between −41 and −60 mV); (vii) very good stability (values between −61 and −80 mV); (viii) extremely good stability (values between −81 and −l00 mV) [45].

The zeta potential of our samples was between −23.56 for TOCE and −18.47 for TOE. The lowest value of zeta potential was recorded inpk the ternary emulsion TOCE, while the values for binary emulsions increased in order: OCE < TCE < TOE. Given the range of values for the zeta potential of the obtained emulsions (−18.47 to −23.56) presented in Table 3, it is observed that they can be included in the category of the system with low dispersion. The stability of the emulsion is higher the more negative the values. For these reasons, we can say that the tertiary system ensures maximum emulsion stability, while TOE is the less stable system. Therefore, to stabilise an emulsion, it is necessary to lower its zeta potential.

Another study that includes Pickering emulsions with nanocellulose in the form of nanocrystals (CNC) or nanofibers (CNF) showed zeta potential values of −45.6 ± 3.6 and −49.9 ± 3.9 mV, respectively, and were considered highly electrostatically stable samples [21]. The CNF emulsions presented significantly higher values than the CNC emulsions (−18.3 ± 1.4 and −11.1 ± 0.9, respectively), characterised as low-dispersion emulsion at the beginning of agglomeration. The study explains the difference based on the chemical composition of the essential oils: the ester as a terminal group and a benzene ring may be responsible for the increase in the negative charges [21].

### 4.3. Antifungal and Antimicotoxicogenic Potential of EO Mixtures and Emulsions

Over the years, efforts have been devoted to the search for new antifungal products from natural sources for food preservation, with several plant extracts being reported for their antifungal activity [46,47,48,49].

Given the limited number of synthetic antifungal agents available, and the fact that most of them have similar modes of activity, their combination with natural antifungals may increase the potential for synergistic interaction [50]. The comparison of the effectiveness against Fusarium of some essential oils and mixtures with synthetic fungicides was previously addressed in other studies [29]. The results highlighted that the mycelia radial growth was similar to thiophanate methyl (as the positive control); the differences were not statistically significant (*p* > 0.05) compared to the positive control [29].

Given this and our previous study regarding the antifungal and antimycotoxicogenic potential of EOs [24], the synergistic potential obtained by the association of EOs in binary/tertiary combinations and emulsions was assessed in this study.

The results showed that the storage of wheat seeds in an atmosphere enriched by EO vapours directly affected the mycobiota of the seeds, especially after 28 days. All binary/tertiary combinations have inhibition potential against fungal development, but the period of action differs depending on the EOs and the strains.

After 7 days of treatment with the binary mixtures OC, TO, and TC, the development of *Fusarium* was inhibited, but the corresponding binary emulsions led to an increase in fungal growth. The control of *Cladosporium* was effective after 14 days for all binary/tertiary EOs and emulsions. For *Alternaria,* the same pattern was observed except when TC was applied to wheat seeds. Moreover, the incidence of *Fusarium* decreased in time after treatment and was minimal after 28 days of treatment.

Based on the results from Figure 1, we can summarise that the maximum effect on *Fusarium* was obtained when exposed to TO, and a higher efficiency was observed in the case of EO treatment compared to emulsions. A possible explanation may be the prolonged exposure of spores/hyphae to EO vapours and the cumulative effect over time that would affect their viability.

A previous study has highlighted the antifungal potential of EOs on the fungal development and mycotoxin production in wheat [4]. In this study, Lovrin 34 wheat was naturally contaminated with 0.689 ppm fumonisin and 0.420 ppm DON and sterilised and treated with several EOs—*Melissa officinalis* L., *Salvia officinalis* L., *Coriandrum sativum* L., *Thymus vulgaris* L., *Mentha piperita* L., and *Cinnamomum zeylanicum* L.—in three different concentrations of each oil (500, 1000, and 2000 ppm). The results obtained highlighted the role of EO_S_ in the control of *Fusarium* sp. and mycotoxins.

In another study, a comparative analysis of the composition and active property of certain EOs, including thyme EOs, was carried out by Vasile et al., 2017 [51]. The study highlighted the highest inhibition potential of EOs against *Fusarium graminearum* compared to other EOs. Moreover, the antifungal potential of coriander EO against *Fusarium culmorum* was recently reported [38]. *Origanum vulgare* EO activity against different *Fusarium* and *Penicillium* sp. was analysed in wheat seeds [52,53]. Further relevant contributions to sustainable disease control using EOs were reported in the extensive study by Abubakar AI, 2022 [54].

The antifungal and antimycotoxicogenic effects of EOs and natural emulsions based on EOs can be attributed to their chemical composition and the interaction between their components. Monoterpenes, the main chemical constituents of *Coriandrum sativum* EO obtained from ripe fruit, contain a high percentage of linalool (69.8–87.54%), which can be used as a potential antifungal agent in stored grains [55]. By applying *Coriandrum sativum* EO, extremely significant results were obtained regarding the fungal growth, regardless of concentration.

The correlation analysis (Table 5) showed a strong influence of the four major EO compounds (linalool, carvacrol, thymol, and cymene) on the inhibition percentage of DON development.

Oregano EO is active against moulds, especially aflatoxin-producing strains and foodborne bacteria [56,57]. Oregano EO exhibited a fungicidal effect due to a chemical composition rich in phenolic compounds, which can cause damage at the mycelial level [57]. It has been demonstrated that oregano EO can be used to prevent infection with *Fusarium* spp. due to the presence of an aromatic nucleus and a phenolic OH group, as in carvacrol, the main chemical compound in TO and CO [58]. The mechanism of action refers to the different penetration capacities through the fungi membrane depending on the hydrophobicity of the molecules or even the position of the hydroxyl groups. Carvacrol and thymol have a prominent disintegrating effect on the outer membrane, which could explain the major antimicrobial activity exerted by these two fractions. Cui H. et al., 2019 [22] highlighted that oregano EO can destroy the membrane structure of strain cells, causing leakage of contents, inhibiting intracellular enzyme activity, and affecting the normal physiological metabolism of the cells [22].

The fungistatic and fungicidal potential of *Thymus vulgaris* L. EO was determined in previous studies [47,59] and these actions were associated with the chemical composition of the EO and explained by the alteration of the membrane potential of strain cells [60]. The mode of antifungal action at the cellular level was also investigated. A previous study demonstrated that thyme EO caused significant damage to the cell membrane and induced apoptosis of cells in treated hyphae [61]. This mode of action can be attributed to thymol, the major component of the thyme EO, which would inhibit ergosterol biosynthesis and the disruption of membrane integrity in fungal cells [62].

On the other hand, several studies have shown that EOs have a stronger antimicrobial activity than major constituents or their mixtures, suggesting synergistic effects among their minor components and the importance of all components in relation to the biological activity of EOs [47,63,64].

Our work agrees with previous studies regarding the antifungal effect of oregano, coriander, and thyme EOs [26,31,32,34,39]. In our experiment, it should be noted that the maximum effect on fungi and DON production was observed with the mixture of thyme and oregano EOs, the effect maximised on fungal growth when using TO and on DON when using TOE. This would indicate that excipients such as lecithin added in the emulsion preparation can improve the antimycotoxicogenic effects of EOs. A similar pattern was noted by other researchers who reported that the mycotoxin inhibitory activity of thyme EO was boosted when incorporated in nanoemulsion [18].

### 4.4. Physical–Chemical Analysis of Wheat Seeds

Recognising the physical–chemical changes in wheat seeds after fungal exposure is very important in the bakery industry. Moreover, the influence of antifungal and antimycotoxicogenic treatments on the nutritional value of wheat and the flour obtained are critical issues in the bakery industry. It is known that storage conditions, especially temperature and humidity, contribute to the growth, survival, and development of fungi and affect their ability to produce mycotoxins. For these reasons, it is important to pay more attention to administering treatments with natural antifungal products to prevent their negative influence on the nutritional content of wheat.

The humidity of wheat seeds was around 16% in all samples, without statistical differences between treatments and the control. The optimal conditions for the biosynthesis of mycotoxins in the grain are met at a temperature of 25–32 °C and 16–30% humidity. Postharvest management significantly mitigates mycotoxins and controls wheat contamination in food chains during harvesting, cleaning, drying, storage, and processing. Temperature and humidity control up to the safe storage phase was a key point for attenuating the concentration of mycotoxins in cereal grains [10].

In our experiment, the protein content varied between 13.3 and 13.9% in all samples treated with EOs and emulsions, with significant differences recorded between OC (where the highest protein value was registered) and other EOs. It should be noted that the treatment application did not lead to a decrease in the protein content in the wheat samples; on the contrary, the protein values were stimulated, especially in the case of emulsions. The explanation could be given by the inversely proportional correlation of the protein content with the mycotoxin intake.

The treatment with OC produced a lower inhibition rate (37.52%). In this context, we can associate the higher DON accumulation with the increased protein content in wheat seeds. The same pattern was reported by other researchers [65,66] in recent studies. They observed that the protein content increased in the presence of Fusarium artificial inoculation. The cause of the protein increase in the wheat sample affected by DON contamination could be the decrease in carbohydrates used by the developing pathogens [18]. However, other authors observed no effect on the protein level after *Fusarium* infection [18,67] or a decrease in this chemical parameter [68].

To our knowledge, no studies regarding the influence of *Fusarium* sp. or DON contamination on the elemental composition of wheat have been reported. However, it was mentioned that the flavonoid content increased in the inoculated winter wheat with *Fusarium* and was lower in the samples treated with fungicides [69]. Significant differences between the control and all the trials with EO and emulsion treatment were observed regarding the mineral content. All the binary/tertiary combinations exhibited higher mineral content in wheat seeds compared with the control, and the enhancement of the nutritional value of bakery wheat.

Regarding the colour of wheat seeds, this parameter can be affected by treatment with EOs depending on the physical appearance of the EOs and emulsions. Significant differences related to the control were observed after exposure to binary/tertiary EOs and emulsions, which may influence the sensory properties of the flour obtained after treatment. Further studies on the influence of EO treatment on the taste/smell/colour of flour and baked wheat products are needed.

## 5. Conclusions

The results obtained in this study show the possibility of using binary/tertiary mixtures of EOs and emulsions based on oregano, coriander, and thyme EOs as healthy and environmentally friendly alternatives to synthetic fungicides in the wheat-processing industry. The natural mixtures obtained as oil-in-water (O/W) emulsions and stabilised using lecithin as an emulsifier represent stable systems with low dispersion capacity, capable of being used in grain warehouses and bakeries as antifungal and antimycotoxicogenic agents.

Regarding antifungal potential, the results showed that the storage of wheat seeds in an atmosphere enriched by EO vapours directly affects the mycobiota of the seeds, especially after 28 days. All binary/tertiary combinations exhibited inhibitory potential on fungi and DON production. The antifungal and antimycotoxicogenic effects were maximum when the mixture based on thyme and oregano was applied to the wheat seeds, with the binary combination more active against fungi and the binary emulsion more effective against DON biosynthesis.

It should be noted that the tertiary combination, of both EOs and emulsions, did not lead to a maximum antifungal and antimycotoxicogenic effect, indicating the occurrence of antagonistic effects between the three EOs that influence the action potential against fungi and DON development.

Using binary and tertiary mixtures as antifungal agents on wheat seeds destined for bakery influences the physical–chemical parameters of the seeds. Moreover, an increase in protein and mineral content with beneficial effects on the nutritional value of wheat and changes in the colour of the samples were observed.

## Figures and Tables

**Table 1:** The composition of EOs/emulsions used in the wheat seed treatment.

Type of EOs/Emulsions *		Composition				
	Abbreviation	Coriander (µL)	Oregano (µL)	Thyme (µL)	Lecithin (mg)	Water (mL)
EOs O/C	OC	100	100	-	-	-
EOs T/O	TO	-	100	100	-	-
EOs T/C	TC	100	-	100	-	-
EOs mixture based on T/O/C	TOC	100	100	100	-	-
Binary emulsion O/C	OCE	600	200	-	6	99.2
Binary emulsion T/O	TOE.	-	200	600	6	99.2
Binary emulsion T/C	TCE	600	-	600	6	98.8
Tertiary emulsion T/O/C	TOCE	600	200	600	6	98.6

* EOs O/C (mixture based on oregano and coriander); EOs T/O (mixture based on thyme and oregano); EOs T/C (mixture based on thyme and oregano); EOs T/O/C (mixture based on thyme, oregano, and coriander); binary emulsion O/C (binary emulsion based on oregano and coriander); binary emulsion T/O (binary emulsion based on thyme and oregano); binary emulsion T/C (binary emulsion based on thyme and coriander); tertiary emulsion T/O/C (tertiary emulsion based on thyme, oregano, and coriander).

**Table 2:** Chemical composition of binary and tertiary mixtures of EOs *.

		% of Total Compounds			
Compounds	Retention Time	TO	TC	OC	TOC.
α-Pinene	11.65	2.07	5.07	5.19	4.62
Camphene	13.15	0.54	0.84	0.19	0.55
β-Pinene	14.11	1.58	1.85	0.37	1.44
β--Myrcene	15.41	2.33	1.08	2.14	2.41
D-Limonene	16.22	1.86	3.16	3.54	3.35
γ-Terpinene	17.64	nd	nd	nd	nd
terpinolene	18.50	0.23	0.11	0.39	0.31
Eucalyptol	18.67	0.26	0.29	0.07	0.23
o-Cymene	20.07	**33.25**	**29.33**	**24.35**	**14.59**
β-Linalool	27.67	3.48	**28.87**	**28.22**	**21.68**
Anisole	28.93	0.54	0.69	0.01	0.47
Thymol-methyl-ether	29.70	0.35	nd	nd	0.30
Borneol acetate	29.82	0.13	0.18	0.02	0.12
4-Terpineol	30.45	0.41	nd	nd	0.36
Caryophyllene oxide	42.07	0.85	0.53	0.54	0.81
Thymol	43.65	**21.04**	**26.18**	0.35	**17.22**
Carvacrol	44.65	**30.33**	1.63	**33.72**	**30.69**
Total		**99.25**	**99.81**	**99.10**	**99.15**

* OC—binary mixture of oregano and coriander EOs, TO—binary mixture of thyme and oregano EOs; TC—binary mixture of thyme and coriander EOs; TCE—binary emulsion of thyme and coriander EOs; TOC—tertiary mixture of oregano, coriander, and thyme EOs; nd—not detectable.

**Table 3:** Mean particle size (nm), polydispersity index (PDI), and zeta potential (ζ-Potential) (mV) of natural preparations.

Natural Preparations *	Size and Particle Homogeneity			Zeta Potential (ζ-Potential) (mV)
	Mean Particle Size (nm)	The Proportion of Each Population (%)	The Polydispersity Index (PDI.)	
OCE	617.1 883.4	23 77	0.5	−22.72
TCE	723.9	100	0.3	−21.45
TOE	583.3 701.8	48 52	0.6	−18.47
TOCE	570.7 887.6	63 37	0.6	−23.56

* OCE—binary emulsion of oregano and coriander EOs; TOE—binary emulsion of thyme and oregano EOs; TCE—binary emulsion of thyme and coriander EOs; TOCE—tertiary emulsion of oregano, coriander, and thyme EOs.

**Table 4:** Fungistatic and fungicidal effect of the analysed EOs on *Fusarium graminaerum* expressed as minimum concentration with fungistatic effect (CMFs) and minimum fungicidal concentration (CMFg).

EOs	CMFs (%)	CMFg (%)
*Coriandrum sativum* (coriander)	0.5	-
*Thymus vulgaris* (thyme)	0.1	0.6
*Origanum vulgare* (oregano)	0.06	0.2

**Table 5:** Pearson correlation coefficient matrix for DON content and DON inhibition (%) and the chemical composition of EO mixtures (TO, TC, OC).

	DON Content	DON Inhibition	α-Pinene	β-Pinene	β-Myrcene	D-Limonene	o-Cymene	β-Linalool	Thymol	Carvacrol
DON content	1									
DON inhibition	−1	1								
α-Pinene	−0.618	0.618	1							
β-Pinene	−0.023	0.023	−0.376	1						
β--Myrcene	−0.162	0.162	−0.590	−0.526	1					
D-Limonene	−0.530	0.530	0.983	−0.539	−0.433	1				
o-Cymene	−0.928	**0.928**	−0.848	0.810	0.073	−0.931	1			
β-Linalool	−0.865	**0.865**	0.990	−0.238	−0.700	0.947	−0.763	1		
Thymol	−0.710	**0.710**	0.372	0.720	−0.969	0.196	0.176	0.502	1	
Carvacrol	−0.983	**0.983**	0.812	−0.846	−0.008	0.905	−0.998	0.719	−0.240	1

## Data Availability

The report of the analysis performed for the samples in the paper can be found at the Interdisciplinary Research Platform (PCI) belonging to the Banat University of Agricultural Sciences and Veterinary Medicine “King Michael I of Romania” from Timisoara.
